# Automated measurement of Little’s Irregularity Index on intraoral photographs using a convolutional neural network

**DOI:** 10.1186/s12903-026-09870-7

**Published:** 2026-09-16

**Authors:** Moritz Kanemeier, Tuna Ergün, Thomas Stamm, Claudius Middelberg, Jonas Q. Schmid

**Affiliations:** Department of Orthodontics, University of Münster, Albert-Schweitzer-Campus 1, Gebäude W 30, 48149 Münster, Germany

**Keywords:** Orthodontics, Tooth displacement, Photography, dental, Artificial intelligence, Deep learning, Machine learning

## Abstract

**Background:**

Quantification of dental irregularities is essential for assessing case severity, evaluating treatment outcomes, and aiding early detection of fixed retainer failure. Measurements are typically performed on casts or digital scans, although manual measurement on intraoral photographs has been explored to enable remote monitoring. This study evaluated automated measurement of mandibular anterior irregularity on intraoral photographs using a deep learning model.

**Methods:**

A dataset of intraoral occlusal photographs annotated with contact points of mandibular anterior teeth was created to train a neural network model. Individual tooth displacements and Little’s Irregularity Index (LII) were computed from these contact points. Annotation reliability was assessed using intraclass correlation coefficients, and agreement between model-derived and reference LII measurements was evaluated using Bland-Altman analysis on an independent test set. A supplementary, exploratory Bland-Altman analysis was performed at the tooth level. An a priori power analysis determined the required size of the test set, based on a predefined equivalence threshold of 2 mm for LII.

**Results:**

The model demonstrated precise localisation of contact points, with a mean radial error of 0.55 ± 0.22 mm. Tooth displacement and LII were predicted with mean absolute errors of 0.42 ± 0.32 mm and 1.37 ± 0.89 mm, respectively. For LII, Bland-Altman analysis revealed a significant bias of 1.07 mm, with limits of agreement ranging from -1.36 mm to 3.50 mm. The exploratory tooth-level analysis yielded a bias of 0.21 mm and descriptive limits of agreement ranging from -0.74 mm to 1.17 mm.

**Conclusions:**

The neural network enables automated detection of mandibular anterior landmarks and estimation of LII from intraoral photographs. Since the limits of agreement were wider than the predefined equivalence threshold, the proposed model can currently only be recommended as a complementary tool.

## Background

Orthodontic indices are essential tools for objective assessment of case severity and evaluation of treatment outcomes, providing standardised measures of alignment and occlusion. 

For the assessment of mandibular anterior tooth misalignment, the Irregularity Index was introduced by Robert M. Little in 1975 [1]. The index sums the linear displacements between adjacent contact points of the six mandibular anterior teeth, providing a single score that reflects the severity of misalignment.

Traditionally, Little’s Irregularity Index is measured manually on dental casts using a calliper to determine interproximal contact point displacement. Although widely used in research and clinical practice, this method has been criticised for poor inter-examiner reproducibility [2]. Digital model analysis has addressed some of these limitations by reducing subjectivity in defining the measurement plane and eliminating the technical challenges of calliper-based measurements [3–5]. However, these approaches still rely on impressions or intraoral scans, limiting their practicality for routine monitoring, large-scale epidemiological studies, and remote assessment.

Two-dimensional intraoral photographs represent a promising alternative, as they can be easily acquired without specialised equipment. They may also improve patient comfort, particularly in individuals with a sensitive gag reflex, and allow simultaneous documentation of enamel conditions such as developmental defects, molar-incisor hypomineralisation (MIH), and white spot lesions [6, 7]. Previous studies have shown that manual measurement of Little’s Irregularity Index on photographs can be reliable and reproducible [8, 9]. However, manual annotation remains challenging due to variations in image quality and limited visibility of interproximal landmarks.

Recent advances in deep learning have enabled accurate automated analysis of dental and craniofacial structures from two-dimensional radiographs [10–14]. These developments suggest potential for automated landmark detection on intraoral photographs, allowing direct estimation of tooth alignment without impressions or scans. To date, only a few studies have explored automated landmark detection on intraoral photographs [15, 16]. In particular, Hertig et al. demonstrated the feasibility of automated quantitative tooth-crowding analysis using a convolutional neural network [16]. The present study extends this work by evaluating an alternative network architecture and by assessing agreement between automated and manual LII measurements against a prospectively defined clinical equivalence threshold in a separate test set whose size was determined by an a priori sample-size calculation.

The aim of this study was to develop and evaluate a deep learning model for the automatic measurement of Little’s Irregularity Index on intraoral photographs. The focus was on evaluating the model’s clinically relevant performance using predefined equivalence limits and a sufficiently powered Bland-Altman analysis. The null hypothesis tested was that the 95% limits of agreement (LoA) would exceed the predefined equivalence limit, indicating insufficient agreement between AI-based and manual measurements.

## Methods

### Dataset

Intraoral occlusal photographs of patients treated with multibracket appliances in the Department of Orthodontics of the University Hospital Münster over the last five years were randomly selected.

Inclusion criteria were that at least one mandibular intraoral photograph was available, showing all permanent mandibular incisors, canines and first molars. Exclusion criteria were the presence of supernumerary teeth in the mandibular anterior region.

All photographs were acquired under routine clinical conditions following standard intraoral photography protocols.

A single trained examiner manually annotated the mesial and distal contact points of the mandibular incisors, canines, and first molars on each image using custom software [17], with intrarater reliability verified by ICC analysis. These annotations were used for both model training and evaluation.

The dataset was split patient-wise into training, validation and test subsets. The training subset was used for model fitting and cross-validated hyperparameter optimisation. The validation set was used to monitor performance during the final model training to apply early stopping and select the final model checkpoint. The test set was held out exclusively for the final statistical evaluation.

The sufficiency of the training subset was confirmed by learning-curve analysis and the size of the test subset was determined a priori by power analysis for the Bland-Altman agreement evaluation.

### Neural network architecture

A convolutional neural network based on the High-Resolution Network (HRNet) architecture was employed for landmark detection [18]. The HRNet-W32 variant, pretrained on ImageNet [19], was selected.

**Fig. 1:**
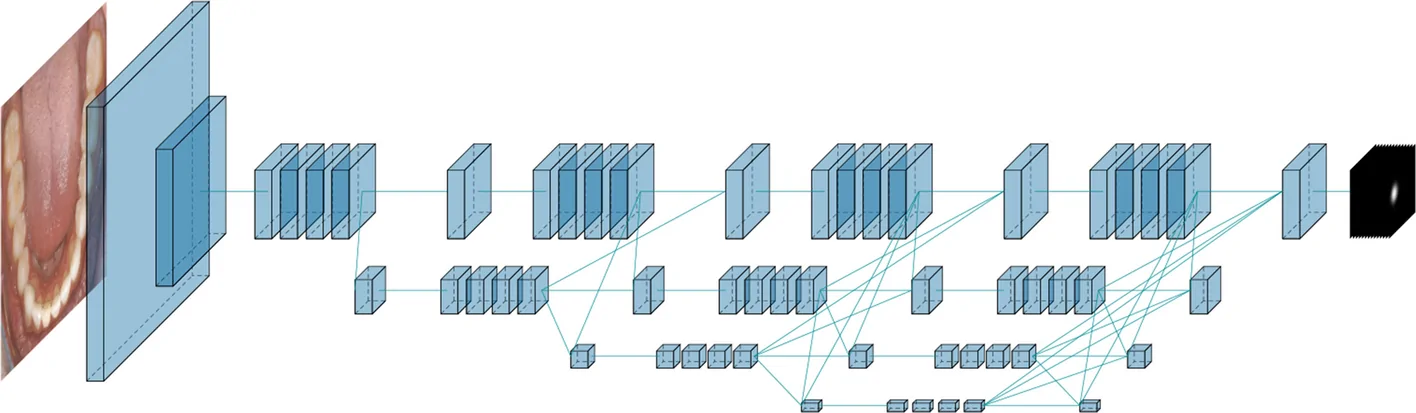
Schematic illustration of the network architecture. The mandibular intraoral images are processed by the High-Resolution Network (HRNet, [18]) architecture. This architecture processes the images in multiple stages, adding more branches at each stage. The initial high-resolution branch is supplemented by additional, lower-resolution branches that offer a higher level of abstraction. After each stage, information is exchanged between all branches, enabling the network to preserve fine spatial detail while enriching the semantic context. Finally, the network outputs one heatmap per landmark, representing the probability of its presence at each pixel

The network takes intraoral photographs as input and outputs one heatmap per landmark, corresponding to the mesial and distal contact points of the mandibular incisors, canines, and molars (Fig. 1). Each heatmap represents the likelihood of a landmark at each pixel, modelled as a Gaussian distribution centred at the ground-truth position.

Training used an equally weighted composite loss combining mean squared error on heatmaps and a coordinate-based L1 loss.

### Hyperparameter optimisation

Five-fold patient-wise cross-validation on the training set was used to optimise learning rate, weight decay, and augmentation strength. The optimal configuration was then used to retrain the model on the full training set.

### Training procedure

Images were resized and normalised prior to training. Data augmentation included random rotation, scaling, and translation.

Optimisation was performed using adaptive moment estimation (Adam; [20]) with default parameters. Training ran for up to 500 epochs with a batch size of 12, using early stopping after 20 epochs without validation loss improvement. The final model corresponded to the checkpoint with the lowest validation loss.

Implementation was carried out in PyTorch 2.9.0 with Torchvision 0.24.0 on a workstation running Ubuntu 24.04 (Intel Core i9-10900K CPU, 128 GB RAM, NVIDIA RTX 3080 Ti GPU with 12 GB VRAM).

### Computation of Little’s Irregularity Index

Following landmark prediction, Euclidean distances between adjacent contact points of the mandibular incisors and canines were calculated in pixel coordinates.

Distances were converted to metric units using an image-specific scale factor derived from mesial and distal molar landmarks and the average mesio-distal molar width from a recent meta-analysis [21].

The Little’s Irregularity Index for each image was calculated as the sum of the scaled distances.

### Statistical analysis

All final model-performance and agreement analyses were conducted exclusively on the independent test set, which was not used for model development or selection.

Model performance was evaluated using mean radial error (MRE) between predicted and annotated landmarks. Accuracy of tooth displacements and Little Indices was assessed using mean absolute error (MAE).

Agreement for Little’s irregularity index was evaluated using Bland-Altman analysis at the image level [22]. Proportional bias was assessed via linear regression of differences (AI - manual) against their mean. Agreement was defined according to a predefined equivalence margin (*δ ~=~2.0* mm), requiring the 95% confidence intervals of the limits of agreement to lie within $\pm ~\delta$ [23].

A supplementary, exploratory Bland–Altman analysis was also performed at the tooth level to characterise local measurement differences and facilitate comparison with previous work. Because multiple tooth-level measurements were nested within each image and therefore were not independent, this analysis was treated as descriptive and not used for hypothesis testing.

An a priori power analysis based on published estimates [16] determined the required sample size of the independent test set following the method by Lu et al. [23].

Intrarater reliability of the manual annotations was evaluated using images of ten randomly selected patients re-annotated after three months. The intraclass correlation coefficient (ICC) for absolute agreement was calculated using a two-way mixed-effects model [24] for both the individual tooth displacements and the image-wise Little Indices. Reliability was interpreted as poor (< 0.5), moderate (< 0.75), good (< 0.9), or excellent (*≥* 0.9) [25].

All analyses were conducted in R (version 4.4.2; [26]) using the blandr (version 0.6.0; [27]) and irr (version 0.84.1; [28]) packages.

## Results

### Dataset and learning curve

A total of 100 patients (43 males, 57 females; mean age 20.7 years, range 9.6–61.6 years) contributed 281 mandibular intraoral occlusal photographs taken at various stages of treatment with and without multibracket appliances in situ to the development dataset.

**Fig. 2:**
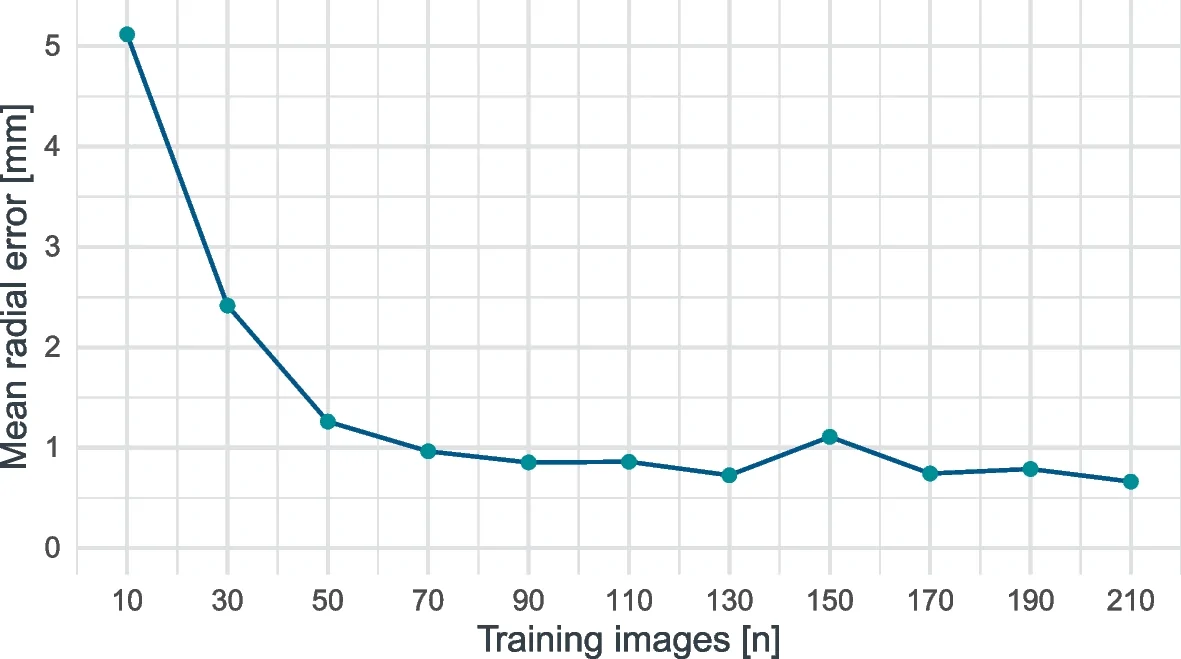
Learning curve of the HRNet model. The mean radial error decreases as the amount of training data increases, reaching a plateau at around 90 images. This confirms that the training dataset size is sufficient

At the patient level, the development dataset was divided into a training set of 80 patients (225 images) and a validation set of 20 patients (56 images). Learning-curve evaluation showed performance plateauing at around 90 training images, indicating that the training set was sufficiently large (Fig. 2).

Power analysis indicated that a minimum of 56 images was required for 90% power in Bland-Altman agreement evaluation. Accordingly, an independent test set was constructed from 60 images of 60 patients (29 male and 31 female; mean age, 19.5 years; range, 10.8–61.9 years), none of whom contributed images to the training or validation sets.

### Hyperparameter optimisation

Hyperparameters were optimised using five-fold patient-wise cross-validation on the training subset. Among the tested learning rates between $10^{-3}$ and $10^{-5}$, a rate of $10^{-4}$ achieved the best validation performance (loss = 2.41 ± 0.55, MRE = 0.85 ± 0.25). Weight decay rates between $10^{-3}$ and $10^{-5}$ showed no improvement in validation performance. Data augmentation was evaluated across four configurations (none, low, medium and high) with progressively increasing strength, including rotations of up to $\pm ~6^\circ$, scaling up to $\pm ~15~\%$, and translations up to $\pm ~6~\%$. The medium augmentation configuration achieved the best validation performance (loss = 2.38 ± 0.55, MRE = 0.85 ± 0.27).

The final model was trained on the entire training subset for 234 epochs using a learning rate of $10^{-4}$, zero weight decay, and the medium augmentation configuration.

**Fig. 3:**
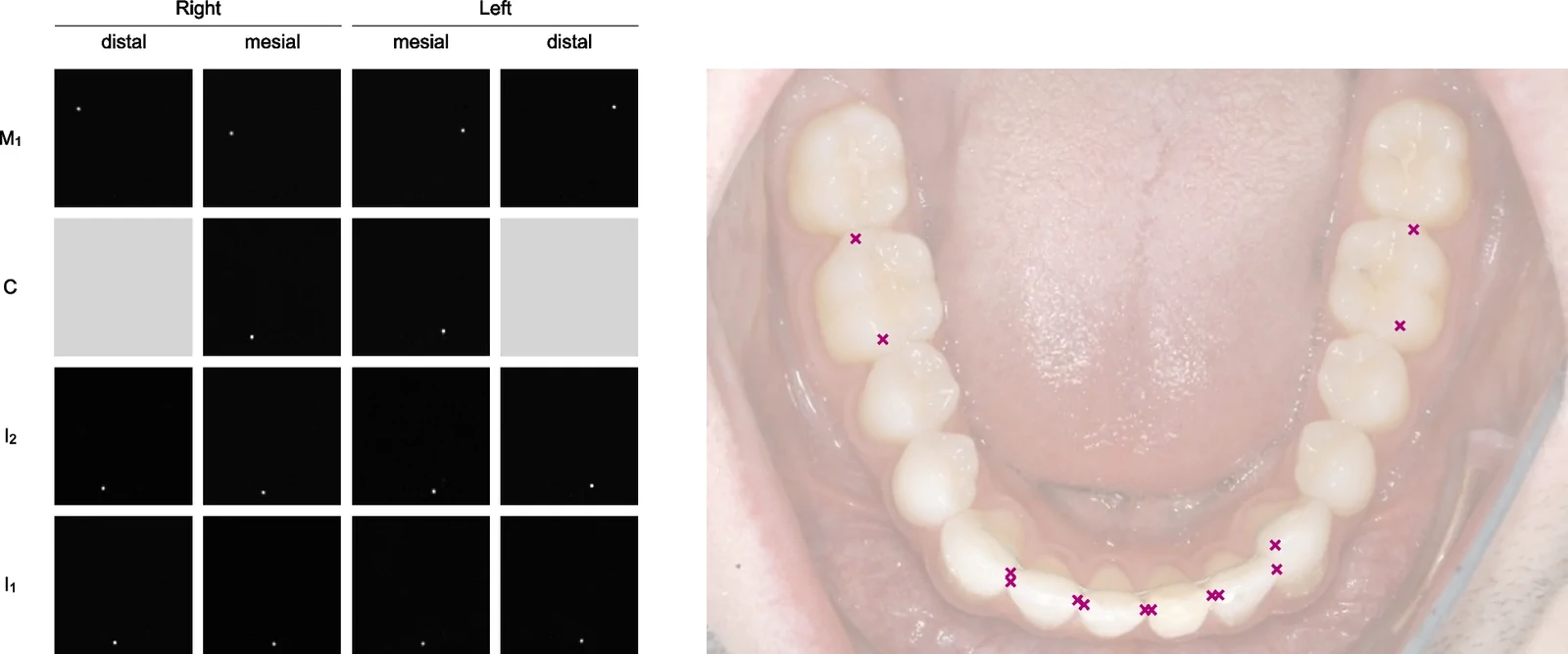
Representative example of predicted heatmaps and corresponding landmark positions. The heatmaps (left) are arranged in rows by tooth type (central incisor ($I_1$), lateral incisor ($I_2$), canine (*C*), and first molar ($M_1$)), and in columns by jaw side (right, left) and contact point (mesial, distal). No predictions are made for the distal contacts on canines. The intraoral image (right) displays the predicted landmark positions overlaid on the intraoral photograph

### Landmark localisation performance

On the independent test set, the model achieved an MRE of *0.55 ± 0.22* mm, with 90% of landmarks localised within 1 mm. A representative example is shown in Fig. 3.

**Fig. 4:**
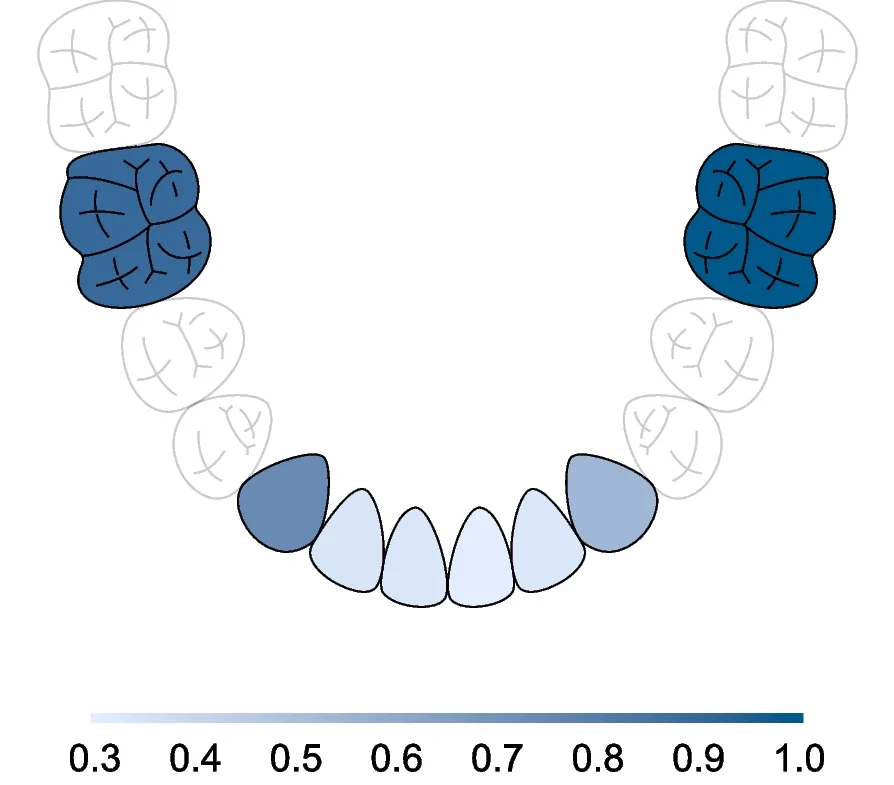
Mean radial error of contact point predictions per tooth. The colour of each tooth indicates the level of error: light blue indicates low error (0.3 mm), while dark blue indicates higher error (1.0 mm). This illustrates that there are slightly higher errors for canines and first molars than for incisors

Tooth-level analysis (Fig. 4) showed the highest accuracy for central incisors (MRE 0.30–0.33 mm), followed by lateral incisors (0.33–0.34 mm) and canines (0.54–0.74 mm). First molars showed slightly higher errors (0.89–0.99 mm) but remained below 1 mm. Overall, posterior and lateral teeth were more challenging to localise.

### Tooth displacements

Tooth displacements ranged from 0.00 to 8.38 mm (mean 1.22 ± 0.99 mm) in the training and validation sets and from 0.00 mm to 5.22 mm (mean 0.92 ± 0.77 mm) in the independent test set. Intrarater reliability of the manual annotations was good (ICC = 0.86). The AI achieved an MAE of 0.42 ± 0.32 mm.

**Fig. 5:**
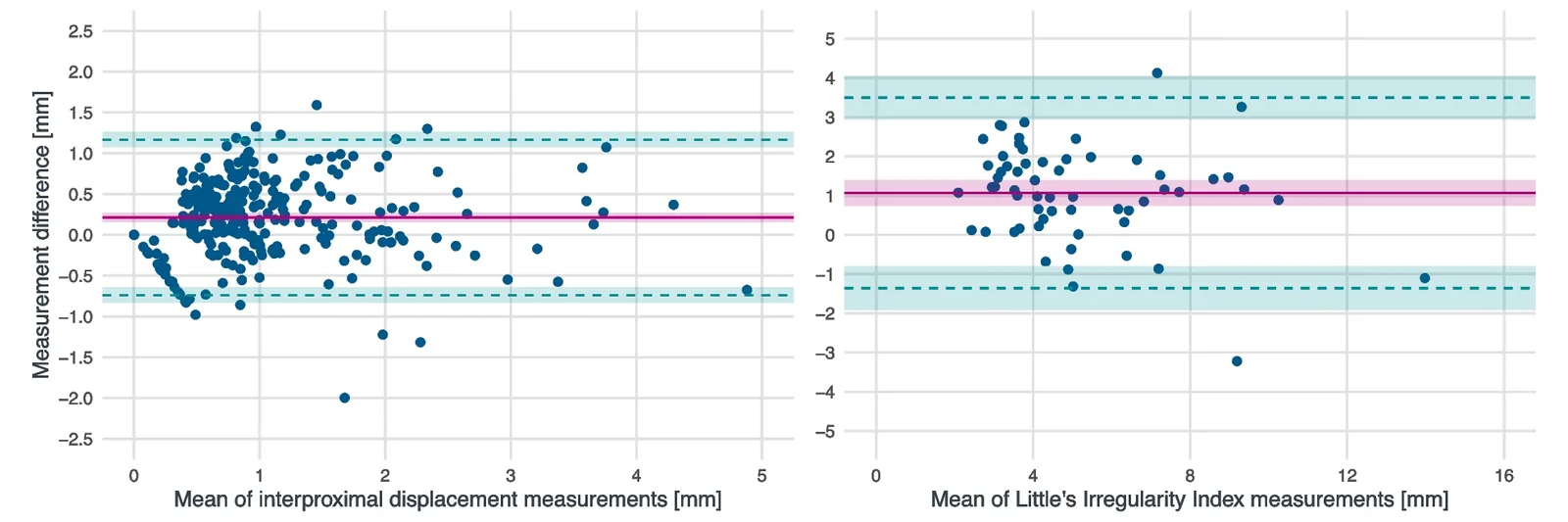
Bland-Altman plots comparing AI-based and manual measurements of tooth-level **interproximal displacement measurements (left)** and image-wise **Little’s Irregularity Index measurements (right)**. Each point represents the difference between the AI-based and manual measurements plotted against their mean, with positive values indicating an overestimation and negative values indicating an underestimation by the AI. The solid pink line indicates the mean bias, and the dashed teal lines represent the 95% limits of agreement (LoA). The shaded bands represent the respective 95% confidence intervals of bias (light pink) and LoA (light teal)

The exploratory Bland-Altman analysis showed a constant bias of 0.21 mm and no proportional bias, indicating that the AI slightly overestimated tooth displacements. The 95% LoA ranged from *-0.74* mm to 1.17 mm (Fig. 5, left).

### Little’s irregularity index

Index values ranged from 1.85 mm to 22.2 mm (mean 6.10 ± 3.21 mm) in the training and validation sets and from 1.50 mm to 14.50 mm (mean 4.60 ± 2.53 mm) in the independent test set. Intrarater reliability of the manual annotations was good (ICC = 0.81). The AI achieved an MAE of 1.37 ± 0.89 mm.

Bland-Altman analysis showed a significant constant bias of 1.07 mm (95% CI: [0.75, 1.39], *p < 0.001*) and a non-significant proportional bias (slope = −0.129, *p* = 0.064), indicating that the AI overestimates irregularity. The 95% LoA ranged from *-1.36* mm (95% CI: [−1.91,−0.81]) to 3.50 mm (95% CI: [2.95, 4.05]) (Fig. 5, right).

### Equivalence assessment

Using a predefined equivalence margin of *δ = 2.0* mm for LII, the 95% confidence intervals of the LoA exceeded this threshold (up to 4.05 mm). Therefore, AI-based LII measurements could not be considered clinically equivalent to manual assessment, and the null hypothesis was not rejected.

## Discussion

This study developed and evaluated a deep learning model for automatic measurement of Little’s Irregularity Index on intraoral photographs, using a predefined equivalence limit of ± 2 mm to assess clinical applicability.

The model achieved high landmark localisation accuracy (MRE *≈* 0.6 mm; 90% within 1 mm), with low error in tooth displacement measurements (MAE 0.42 mm) and an index-level MAE of 1.37 mm.

Constant bias was small, indicating systematic overestimation by the AI. No significant proportional bias was found.

Although clinical equivalence could not be established as the limits of agreement exceeded the predefined threshold, the results demonstrate the technical feasibility of automatically estimating Little’s Irregularity Index from two-dimensional intraoral photographs.

The larger discrepancies observed at the index level can be explained by the additive nature of the index, where small landmark errors accumulate across contact points. Tooth-level analysis showed consistently low errors (*<1* mm), with lower accuracy for canines and molars, indicating greater difficulty in detecting posterior and lateral landmarks. As molar landmarks were used for scaling, small localisation errors may propagate to all derived measurements within an image.

Previous studies reported varying landmark detection accuracy depending on imaging modality and network architecture, with reported MREs of 1.06–1.34 mm for dental landmarks [15], and 0.66–1.08 mm with 82% within 1 mm for cephalometric radiographs [12, 29]. A previous study evaluating the Little Index on intraoral photographs did not provide metrics for the landmark detection performance [16]. In comparison, the present model achieved an MRE of *0.55~± ~0.22* mm with 90% of landmarks within 1 mm, indicating competitive performance.

Direct comparison of Little’s Irregularity Index estimation is limited. Hertig et al. [16] reported MAEs of 0.36 mm for tooth displacements and 0.74 mm for the index. In the present study, comparable tooth-level accuracy was achieved, while slightly larger discrepancies at the index level likely reflect differences in dataset composition, annotation consistency, network architecture, and scaling approaches.

Image scaling represents a key methodological difference between studies and directly impacts measurement agreement. Ryu et al. [15] estimated the image scale by assuming a fixed width of the right central incisor, set at 5.26 mm based on a Korean reference population. The present study follows a similar approach by using the distance between the mandibular first molar contact points and an average width derived from a recent meta-analysis [21]. In contrast, Hertig et al. [16] employed an extended YOLOv8 network capable of predicting both the physical size of each tooth and the location of its contact points. While this adaptive scaling approach eliminates the need for predefined anatomical assumptions, it carries the additional risk of systematic mis-scaling if the predicted tooth width is over- or underestimated. The use of a population-average molar width may introduce scaling errors due to individual anatomical variation and perspective distortion. As the same scaling method was applied to both automated and manual photographic measurements, such errors are not fully captured by the agreement analysis.

It is also important to note differences in dataset sizes and study design. Hertig et al. [16] used 125 images (100 for training, 25 for validation), while Ryu et al. [15] used 1,636 images (1,436 training, 200 validation). Neither study reported a priori sample size calculations to ensure adequate statistical power for their analyses. In the present study, the adequacy of the training-set size was assessed by learning-curve analysis, while the test-set size was determined by an a priori sample-size calculation for the Bland-Altman agreement analysis.

### Strengths and limitations

A strength of this study is the use of a prospectively defined clinical equivalence threshold and an a priori sample-size calculation for the agreement assessment. Additional strengths include systematic hyperparameter optimisation, a dataset covering a wide range of irregularities, patient-wise data splitting to prevent leakage, and the use of an independent test set for statistical evaluation. Limitations include the single-centre design and use of a single annotator, which may limit generalisability. Also, extreme cases may be underrepresented in the dataset. The study assessed agreement with manual photographic annotations rather than accuracy against physical casts or digital models. In addition, scaling based on an average molar width may not account for anatomical variation or perspective distortion. Furthermore, the model was limited to mandibular anterior teeth and cannot be assumed to generalise to other regions or indices.

### Clinical implications

This study demonstrates the technical feasibility of AI-based measurement of Little’s Irregularity Index from standard intraoral photographs, with relatively low mean errors at the tooth contact level. Future research based on this approach may eventually help streamline clinical workflows by eliminating the need for physical impressions or digital scans, while also facilitating remote orthodontic assessments.

As the LoA for the aggregated Little’s Index exceeded the predefined clinical threshold, AI-based measurements should currently be considered a complementary rather than a replacement tool for manual evaluation. One potential future application of remote assessment of mandibular tooth irregularities is the early detection of fixed retainer failure. Such an approach could support timely clinical intervention and potentially prevent more severe complications [30–32].

Future research should investigate the generalisability of the approach across multiple centres and populations and explore extending it to other dental regions or indices. Furthermore, studies examining the performance of AI-based measurements under varying image quality conditions and with multiple annotators would strengthen the clinical applicability of this methodology. Once equivalence with manual photographic measurements has been established, future studies should also validate the complete AI-based workflow against physical casts or three-dimensional digital models, including an evaluation of alternative scaling methods.

## Conclusion

A deep learning model based on HRNet can automatically detect mandibular anterior landmarks and measure Little’s Irregularity Index from intraoral photographs. Little’s Irregularity Indices showed LoA that were wider than the defined acceptable threshold of 2 mm and therefore not considered clinically equivalent. These findings demonstrate the technical feasibility of automatically assessing mandibular anterior alignment and provide a foundation for future studies on photography-based index evaluation.

## Data Availability

The data presented in this study are available on reasonable request from the corresponding author.

## References

[1] Little RM. The Irregularity Index: A quantitative score of mandibular anterior alignment. Am J Orthod. 1975;68(5):554–63. 10.1016/0002-9416(75)90086-x. 1059332

[2] Macauley D, Garvey TM, Dowling AH, Fleming GJP. Using Little’s Irregularity Index in orthodontics: Outdated and inaccurate? J Dent. 2012;40(12):1127–33. 10.1016/j.jdent.2012.09.010. 23000526

[3] Dowling AH, Burns A, Macauley D, Garvey TM, Fleming GJP. Can the intra-examiner variability of Little’s Irregularity Index be improved using 3D digital models of study casts? J Dent. 2013;41(12):1271–80. 10.1016/j.jdent.2013.08.020. 24012518

[4] Vorloeper J, Coenen FA, Lang NA, Niederau C, Knaup I, Craveiro RB, et al. Digital analyses of Bolton tooth size ratios and their association to gender, angle class, and other occlusal traits: a study using a partially automated digital 3D model analysis. Eur J Orthod. 2024;46(5):cjae046. 10.1093/ejo/cjae046. 39233488

[5] Lang FA, Lang NA, Vorloeper J, Niederau C, Craveiro RB, Knaup I, et al. Validation of a digital, partly automated three-dimensional cast analysis for evaluation of orthodontic treatment assessment. Head Face Med. 2025;21(1):36. 10.1186/s13005-025-00515-8. 40341057 PMC12060358

[6] Martins DdS, Ionta FQ, Pompermaier Garlet G, Lima RR, Neves AdA, Rios D, et al. Developmental defects of enamel. S. Karger AG; 2024. pp. 10–34. 10.1159/000538850. 39321764

[7] Ozsunkar PS, Özen Du, Abdelkarim AZ, Duman S, Uğurlu M, Demİr MR, et al. Detecting white spot lesions on post-orthodontic oral photographs using deep learning based on the YOLOv5x algorithm: a pilot study. BMC Oral Health. 2024;24(1):490. 10.1186/s12903-024-04262-1. PMC11044306 38658959

[8] Almasoud N, Bearn D. Little’s irregularity index: Photographic assessment vs study model assessment. Am J Orthod Dentofacial Orthop. 2010;138(6):787–94. 10.1016/j.ajodo.2009.01.031. 21130338

[9] Alrasheed WA, Owayda AM, Hajeer MY, Khattab TZ, Almahdi WH. Validity and reliability of intraoral and plaster models’ photographs in the assessment of Little’s Irregularity Index, tooth size - arch length discrepancy, and Bolton’s analysis. Cureus. 2022;14(3):e23067. 10.7759/cureus.23067. 35308184 PMC8920827

[10] Arik SO, Ibragimov B, Xing L. Fully automated quantitative cephalometry using convolutional neural networks. J Med Imaging. 2017;4(1):014501. 10.1117/1.jmi.4.1.014501. PMC5220585 28097213

[11] Schwendicke F, Chaurasia A, Arsiwala L, Lee JH, Elhennawy K, Jost-Brinkmann PG, et al. Deep learning for cephalometric landmark detection: systematic review and meta-analysis. Clin Oral Investig. 2021;25(7):4299–309. 10.1007/s00784-021-03990-w. 34046742 PMC8310492

[12] Chang Q, Wang Z, Wang F, Dou J, Zhang Y, Bai Y. Automatic analysis of lateral cephalograms based on high-resolution net. Am J Orthod Dentofacial Orthop. 2023;163(4):501–8. 10.1016/j.ajodo.2022.02.020. 36528536

[13] Nordblom NF, Büttner M, Schwendicke F. Artificial Intelligence in Orthodontics: Critical Review. J Dent Res. 2024;103(6):577–84. 10.1177/00220345241235606. 38682436 PMC11118788

[14] Yadalam PK, Pawar AR, Natarajan PM, Ardila CM. A novel dual embedding few-shot learning approach for classifying bone loss using orthopantomogram radiographic notes. Head Face Med. 2025;21(1):49. 10.1186/s13005-025-00528-3. 40640850 PMC12247381

[15] Ryu J, Kim YH, Kim TW, Jung SK. Evaluation of artificial intelligence model for crowding categorization and extraction diagnosis using intraoral photographs. Sci Rep. 2023;13(1):5177. 10.1038/s41598-023-32514-7. 36997621 PMC10063582

[16] Hertig G, van Nistelrooij N, Schols J, Xi T, Vinayahalingam S, Patcas R. Quantitative tooth crowding analysis in occlusal intra-oral photographs using a convolutional neural network. Eur J Orthod. 2025;47(3):cjaf025. 10.1093/ejo/cjaf025. 40396639

[17] Kanemeier M, Middelberg C, Stamm T, Albert F, Hohoff A, Schmid JQ. Accuracy and tracing time of cephalometric analyses on a tablet or desktop computer: A prospective study. Head Face Med. 2024;20(1):9. 10.1186/s13005-024-00413-5. 38347578 PMC10860254

[18] Wang J, Sun K, Cheng T, Jiang B, Deng C, Zhao Y, et al. Deep high-resolution representation learning for visual recognition. IEEE Trans Pattern Anal Mach Intell. 2021;43(10):3349–64. 10.1109/tpami.2020.2983686. 32248092

[19] Deng J, Dong W, Socher R, Li LJ, Li K, Fei-Fei L. ImageNet: A large-scale hierarchical image database. In: 2009 IEEE Conference on Computer Vision and Pattern Recognition. IEEE; 2009. pp. 248–55. 10.1109/cvpr.2009.5206848.

[20] Kingma DP, Ba J. Adam: A method for stochastic optimization. 2014. 10.48550/ARXIV.1412.6980.

[21] Thomas J, Kannan A, Kailasam V. Morphological dimension of the permanent dentition in various malocclusion: a systematic review and meta-analysis. BMC Oral Health. 2025;25(1):857. 10.1186/s12903-025-06203-y. 40450279 PMC12125774

[22] Bland JM, Altman DG. Statistical methods for assessing agreement between two methods of clinical measurement. Lancet. 1986;327(8476):307–10. 10.1016/s0140-6736(86)90837-8. 2868172

[23] Lu MJ, Zhong WH, Liu YX, Miao HZ, Li YC, Ji MH. Sample size for assessing agreement between two methods of measurement by Bland-Altman method. Int J Biostat. 2016;12(2). 10.1515/ijb-2015-0039. 27838682

[24] McGraw KO, Wong SP. Forming inferences about some intraclass correlation coefficients. Psychol Methods. 1996;1(1):30–46. 10.1037/1082-989X.1.1.30.

[25] Koo TK, Li MY. A guideline of selecting and reporting intraclass correlation coefficients for reliability research. J Chiropr Med. 2016;15(2):155–63. 10.1016/j.jcm.2016.02.012. 27330520 PMC4913118

[26] R Core Team. R: A language and environment for statistical computing. Vienna; 2024.

[27] Datta D. blandr: a Bland-Altman method comparison package for R. 2017. 10.5281/zenodo.824514.

[28] Gamer M, Lemon J, Singh IFP. irr: Various coefficients of interrater reliability and agreement. 2019. 10.32614/CRAN.package.irr.

[29] Jiang F, Abdulqader AA, Yan Y, Cheng F, Xiang T, Yu J, et al. Deep learning based quantitative cervical vertebral maturation analysis. Head Face Med. 2025;21(1):20. 10.1186/s13005-025-00498-6. 40140932 PMC11938625

[30] Katsaros C, Livas C, Renkema AM. Unexpected complications of bonded mandibular lingual retainers. Am J Orthod Dentofacial Orthop. 2007;132(6):838–41. 10.1016/j.ajodo.2007.07.011. 18068606

[31] Charavet C, Vives F, Aroca S, Dridi SM. “Wire syndrome” following bonded orthodontic retainers: A systematic review of the literature. Healthcare. 2022;10(2):379. 10.3390/healthcare10020379. PMC8871980 35206992

[32] Schmid JQ, Katsaros C, Sculean A, Galletti C, Bettenhäuser-Hartung L, Janssens Y. The effect of lingual orthodontic appliances in the dimensional reduction of labial gingival recession and root prominence caused by wire syndrome in the anterior mandible: a multicenter study. Quintessence Int. 2025;56(4):306–17. 10.3290/j.qi.b5984435. 39976246

[33] Ethik-Kommission Westfalen-Lippe. Antrag für rein retrospektive Datenanalyse. https://www.aekwl.de/fuer-aerzte/ethik-kommission/antragsunterlagen-hilfestellung/retrospektive-studien/. Accessed 16 July 2026.

